# Inhibition of *miR-193a-3p* protects human umbilical vein endothelial cells against intermittent hypoxia-induced endothelial injury by targeting FAIM2

**DOI:** 10.18632/aging.102729

**Published:** 2020-01-29

**Authors:** Qingshi Chen, Guofu Lin, Jianchai Huang, Lida Chen, Yibin Liu, Jiefeng Huang, Shuyi Zhang, Qichang Lin

**Affiliations:** 1 The Second Affiliated Hospital of Fujian Medical University, Licheng 362000, Quanzhou, China; 2 Department of Respiratory and Critical Care Medicine, The First Affiliated Hospital of Fujian Medical University, Taijiang 350005, Fuzhou, China; 3 Department of Respiratory and Critical Care Medicine, Zhangzhou Affiliated Hospital of Fujian Medical University, Xiangcheng 363000, Zhangzhou, China

**Keywords:** miR-193a-3p, obstructive sleep apnea, intermittent hypoxia, FAIM2, endothelial injury

## Abstract

Objective: The functions and molecular regulatory mechanisms of *miR-193a-3p* in cardiac injury induced by obstructive sleep apnea (OSA) are poorly understood. This study aimed to explore the role of *miR-193a-3p* in intermittent hypoxia(IH)-induced human umbilical vein endothelial cells (HUVECs) injury.

Results: In this study, we found that IH significantly decreased viability but enhanced cell apoptosis. Concurrently, the *miR-193a-3p* expression level was increased in HUVECs after IH. Subsequent experiments showed that IH-induced injury was ameliorated through *miR-193a-3p* silence. Fas apoptotic inhibitory molecule 2 (FAIM2) was predicted by bioinformatics analysis and further identified as a direct target gene of *miR-193a-3p*. Interestingly, the effect of *miR-193a-3p* inhibition under IH could be reversed by down-regulating FAIM2 expression.

Conclusion: In conclusion, our study first revealed that *miR-193a-3p* inhibition could protect HUVECs against intermittent hypoxia-induced damage by negatively regulating FAIM2. These findings could advance our understanding of the underlying mechanisms for OSA-related cardiac injury.

Methods: We exposed HUVECs to IH condition; the expression levels of *miR-193a-3p* were detected by RT-qPCR. Cell viability, and the expressions of apoptosis-associated proteins were examined via CCK-8, and western blotting, respectively. Target genes of *miR-193a-3p* were confirmed by dual-luciferase reporter assay.

## INTRODUCTION

Obstructive sleep apnea (OSA), a common sleep disorder, affects a large proportion of the adult population [1, 2]. Intermittent hypoxia (IH) known as the primary characteristic of OSA is a potential key factor leading to the pathogenesis of OSA-related comorbidities, including cardiovascular disease [3], insulin resistance [4] and Alzheimer’s disease [5]. Over the past few decades, an increased risk factor for cardiovascular morbidities has been consistently observed among OSA patients [6, 7]. Furthermore, increasing evidence indicates that patients with OSA often show endothelial dysfunction, which is an early event in the process of cardiovascular disease [8, 9]. In animal OSA models, IH exposures and long-term sleep fragmentation could lead to endothelial dysfunction [10], thereby supporting a potential causal relationship between OSA and endothelial dysfunction. In two previous studies, they clearly indicated that the impairment of endothelial function was restored with improvement of IH exposures [11, 12]. However, the potential mechanisms involved in the occurrence of OSA-induced endothelial dysfunction are still poorly understood.

MiRNAs are a class of small, noncoding RNAs, with the length of 20-26 nucleotides. They regulate gene expression by binding to the 3′ untranslated region (3′-UTR) of target genes, which leads to the reduction of the corresponding genes by degradation of mRNA or inhibition of mRNA translation [13]. Increasing evidence indicates that miRNAs could regulate various physiological and pathological processes, including cell viability, apoptosis, autophagy, and differentiation [14]. Meanwhile, a number of miRNAs are involved and functional in cardiovascular disease, including acute myocardial infarction (AMI) [15], atherosclerosis [16], atrial fibrillation [17] and cardiac hypertrophy [18]. For instance, *miRNA-214* was highly expressed in elderly AMI patients, which may regulate myocardial cell apoptosis via inhibiting *miR-214* target genes expression [15]. Recently, *miR-193a-3p* has been verified as a key regulator in the development of numerous cancers such as non-small cell lung cancer [19], colorectal cancer [20] and bladder cancer [21]. However, the effects and modulatory mechanism of *miR-193a-3p* in protecting human umbilical vein endothelial cells (HUVECs) from IH-induced apoptosis have not been studied.

In the present study, we first used an in vitro model of endothelial injury induced by IH to investigate the role of and interaction between *miR-193a-3p* and Fas apoptotic inhibitory molecule 2 (FAIM2) in regulating IH-induced endothelial damage. We found that intermittent hypoxia induced endothelial injury in vitro, which was accompanied by the upregulation of *miR-193a-3p*. Inhibition of *miR-193a-3p* attenuated intermittent hypoxia-induced endothelial injury by regulating apoptosis via down-regulating FAIM2 expression. Our novel insights into miRNA functions will elaborate the effects of *miR-193a-3p* in preventing IH-mediated endothelial injury by negatively regulating FAIM2, with the goal of providing new treatments for OSA-related cardiovascular diseases.

## RESULTS

### IH-induced endothelial damage in HUVECs

**Figure 1:**
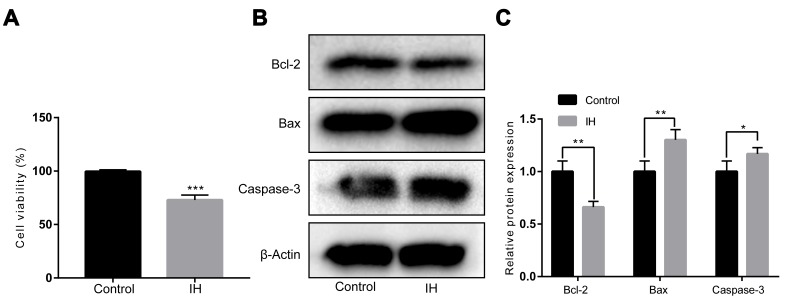
**IH inhibits cell viability in HUVECs.** (**A**) Cell viability by a Cell Counting Kit-8. (**B**, **C**) Western blotting assays for Bcl-2, Bax, and Caspase-3 protein levels. β-Actin was served as internal control. IH: intermittent hypoxia; n = 3. (Data are presented as the mean ± SD of three independent experiments. *P < 0.05, **P < 0.01, and ***P < 0.001).

To evaluate the role of IH conditions for endothelial function, cell viability was detected exposure to normoxia or IH conditions. The results showed that IH treatment significantly decreased cell viability in HUVECs (Figure 1A). Meanwhile, western blot analysis showed that the activities of caspase-3 and the pro-apoptotic protein Bax expression were significantly increased, whereas markedly decreased anti-apoptotic Bcl-2 expression when compared to the normoxia group (Figure 1B and 1C).

### *miR-193a-3p* was upregulated in HUVECs exposed to IH

**Figure 2:**
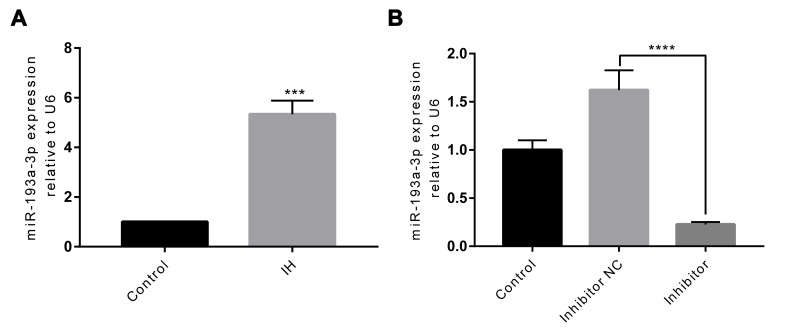
**IH induces upregulation of *miR-193a-3p*, and *miR-193a-3p* is inhibited in HUVECs after transfection.** (**A**) *miR-193a-3p* expression was measured by RT-qPCR. (**B**) Cells were transfected with *miR-193a-3p* inhibitor, and negative control. Relative *miR-193a-3p* expression was normalized to U6. IH: intermittent hypoxia; n = 3. (Data are presented as the mean ± SD of three independent experiments. ***P < 0.001, and ****P < 0.0001).

To assess the effect of *miR-193a-3p* in endothelial function, we first measured the expression levels of *miR-193a-3p* in IH-mediated HUVECs by RT-qPCR. As shown in Figure 2A, *miR-193a-3p* was significantly up-regulated by IH compared to the control group (P < 0.001). Next, to investigate the roles of *miR-193a-3p*, transfection of HUVECs with the *miR-193a-3p* inhibitor, or negative control was further performed. After transfection, the expression of *miR-193a-3p* was determined by RT-qPCR. As expected, *miR-193a-3p* had a remarkable reduction after transfecting with *miR-193a-3p* inhibitor when compared to the negative control group (P < 0.0001; Figure 2B). These outcomes demonstrated that the transfection was efficient.

### *miR-193a-3p* inhibition alleviated IH-induced endothelial injury

**Figure 3:**
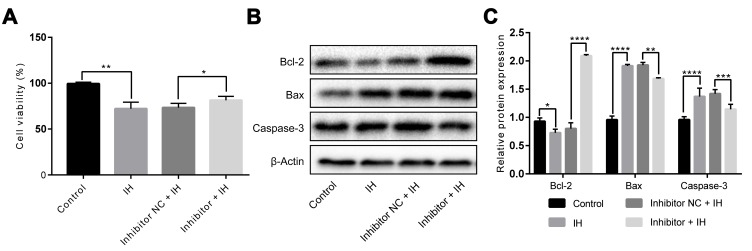
***miR-193a-3p* silence alleviates IH-induced injury in HUVECs.** Cells were transfected with *miR-193a-3p* inhibitor, and negative control. Cells with normoxia treatment were acted as control. (**A**) Cell viability. (**B**, **C**) Expression levels of apoptosis-related proteins. β-Actin was served as internal control. IH: intermittent hypoxia; n = 3. (Data are presented as the mean ± SD of three independent experiments. *P < 0.05, **P < 0.01, ***P < 0.001, and ****P < 0.0001).

To validate if *miR-193a-3p* inhibitor can protect HUVECs from IH-induced injury, we carried out *miR-193a-3p* knockdown experiments. As shown in Figure 3A, results from CCK-8 assay indicated that the cell viability of HUVECs was notably higher than that in the control group after transfecting with *miR-193a-3p* inhibitor (P < 0.05). Additionally, the apoptosis-associated proteins Bcl-2, Bax and Caspase-3 were measured by western blotting. It showed that inhibition of *miR-193a-3p* significantly increased the expression of Bcl-2, whereas markedly decreased Bax and Caspase-3 expression in HUVECs exposure to IH (Figure 3B and 3C).

### *miR-193a-3p* directly targeted FAIM2, and inhibited FAIM2 expression

**Figure 4:**
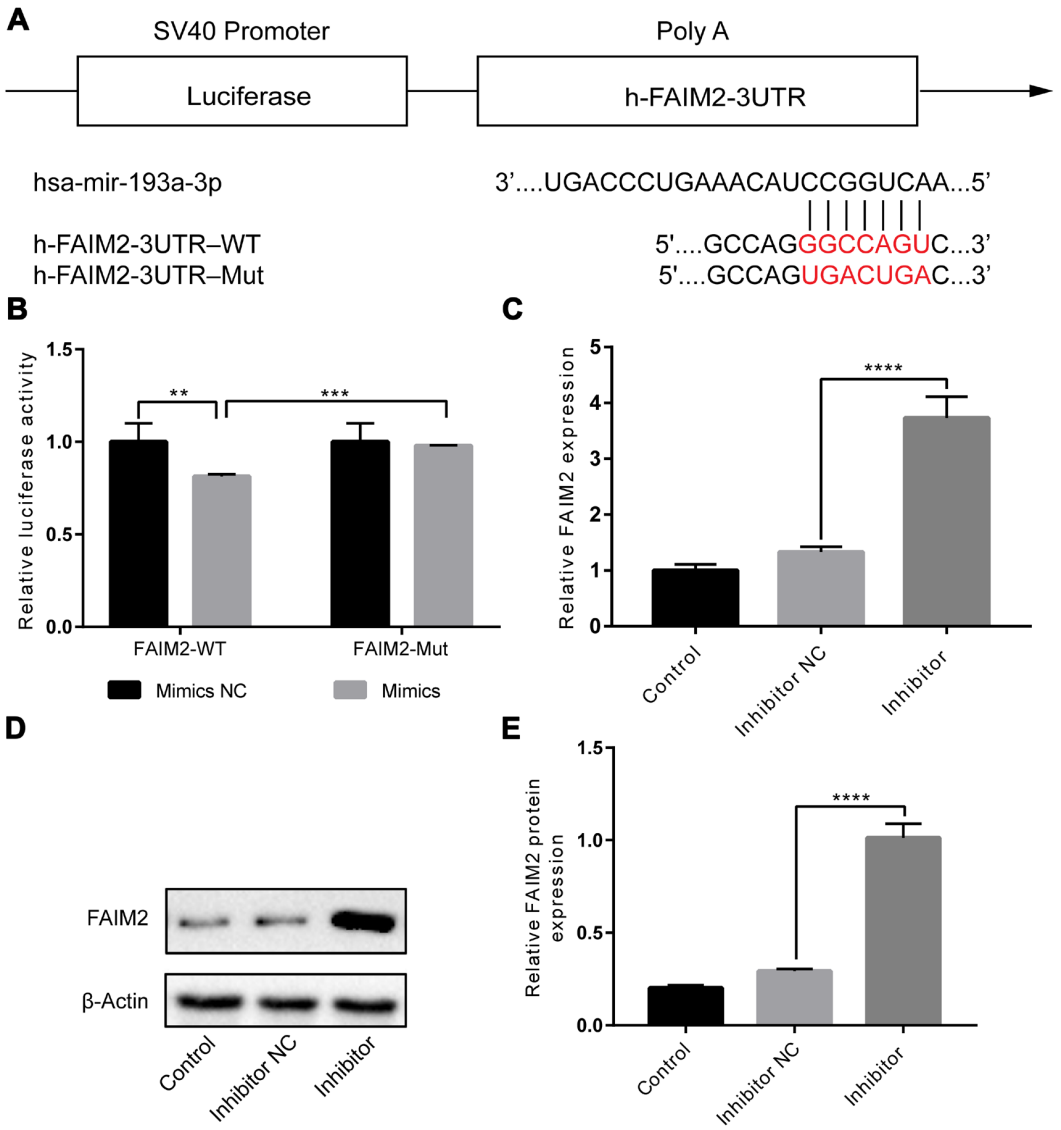
**FAIM2 is a target of *miR-193a-3p*, and FAIM2 could be inhibited by *miR-193a-3p* in HUVECs.** (**A**) The presumptive binding site of *miR-193a-3p* in the 3′-UTR of FAIM2. (**B**) Luciferase reporter assay. We cotransfected HUVECs with wild-type or mutant FAIM2 3′-UTR reporters and *miR-193a-3p* mimics or corresponding control. (**C**–**E**) HUVECs were transfected with *miR-193a-3p* mimics or corresponding control. mRNA and protein expressions of FAIM2 were determined by western blot. n = 3. (Data are presented as the mean ± SD of three independent experiments. **P < 0.01, ***P < 0.001, and ****P < 0.0001).

We carried out bioinformatic analysis to explore the potential mechanism underlying *miR-193a-3p* inhibition suppressed IH-induced endothelial injury. Using miRbase, starBase, and TargetScan, FAIM2 was predicted as a new target of *miR-193a-3p*. The binding site between FAIM2 3′UTR and *miR-193a-3p* is shown in Figure 4A. Next, we performed a dual-luciferase reporter assay to confirm whether *miR-193a-3p* directly targeted to the 3′UTR of FAIM2. As shown in Figure 4B, the results demonstrated that luciferase activity was significantly decreased in HUVECs co-transfected with *miR-193a-3p* mimics and FAIM2-WT compared to that of co-transfection with mimics control and FAIM2-WT. Additionally, the results also revealed that expressions of FAIM2 at mRNA and protein levels were markedly increased by knockdown of *miR-193a-3p* compared to the control group(Figure 4C to 4E). Collectively, these results identified that FAIM2 is a novel direct target of *miR-193a-3p*.

### Knockdown of FAIM2 eliminated the protective effects of *miR-193a-3p* inhibition against IH-induced injury in HUVECs

**Figure 5:**
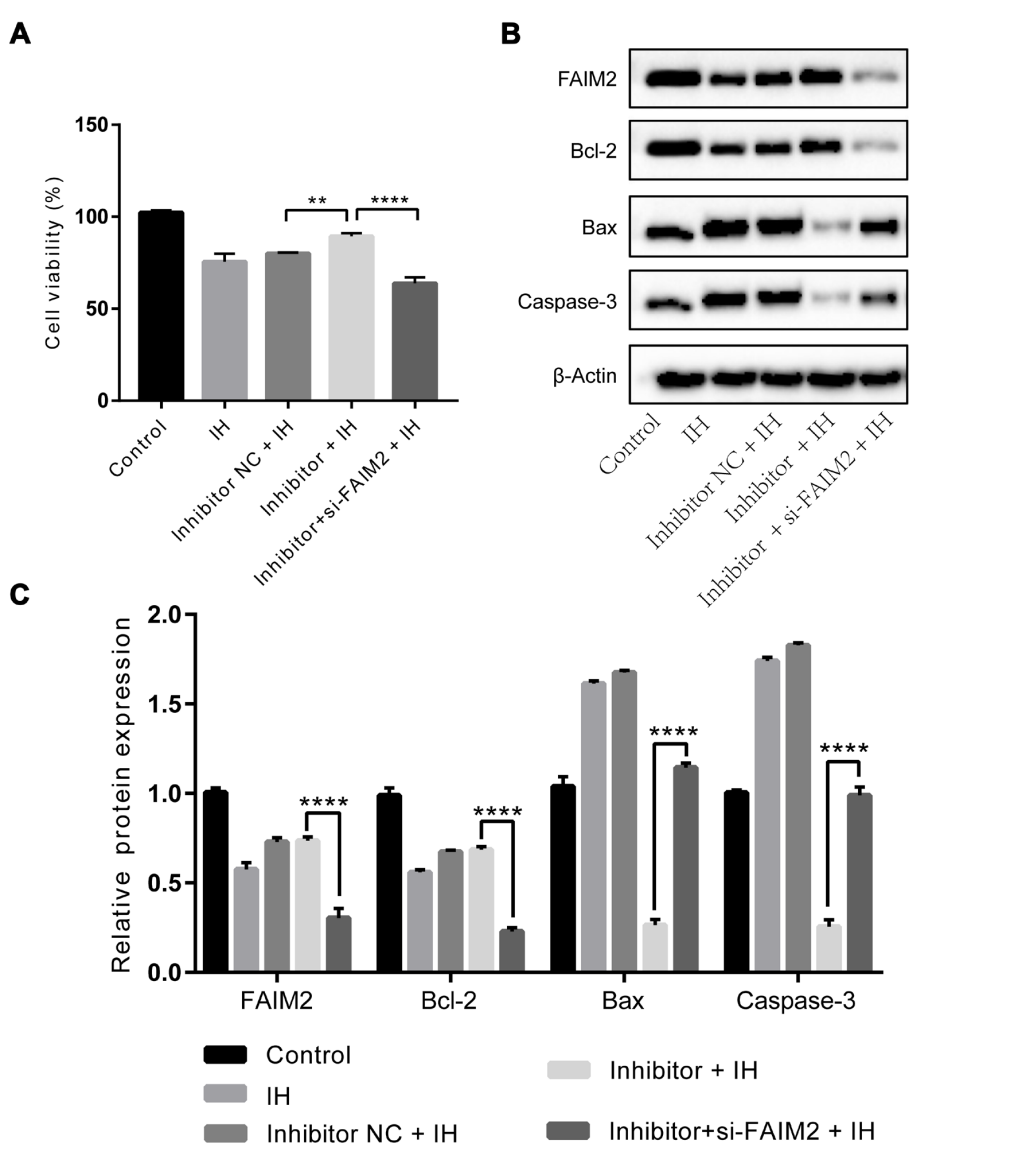
**Effects of *miR-193a-3p* inhibition in HUVECs under IH condition are reversed by knockdown of FAIM2.** *miR-193a-3p* inhibitor, si-FAIM2, and corresponding scrambled control were transfected into HUVECs. Cells without transfection were served as control. (**A**) Cell viability. (**B**, **C**) Western blot assays of FAIM2, Bcl-2, Bax, and Caspase-3 protein. β-Actin was served as internal control. IH: intermittent hypoxia; n = 3. (Data are presented as the mean ± SD of three independent experiments. *P < 0.05, **P < 0.01, ***P < 0.001, and ****P < 0.0001).

Finally, we try to validate whether FAIM2 is linked to the effects of *miR-193a-3p* on IH-induced injury. HUVECs were transfected with si-FAIM2, *miR-193a-3p* inhibitor, or corresponding negative control. As shown in Figure 5A to 5C, the effectiveness of *miR-193a-3p* inhibition on cell viability, and the expression of apoptosis-related proteins were all reversed by knockdown of FAIM2 compared to the control group under IH condition. Therefore, we come up with the conclusion that *miR-193a-3p* silence may ameliorate IH-mediated endothelial injury through up-regulating FAIM2.

## DISCUSSION

In the present study, our data indicated that IH could induced injury in HUVECs and *miR-193a-3p* was remarkably up-regulated under IH condition. However, *miR-193a-3p* inhibitor could protect HUVECs against IH-induced damage, as evidenced by the improvement of cell viability, the down-regulation of Bax, Caspase-3 and the up-regulation of Bcl-2. After that, *miR-193a-3p* was validated to inhibit FAIM2 and FAIM2 was further identified as a novel direct target of *miR-193a-3p* by luciferase reporter assay. Finally, effects of *miR-193a-3p* suppression on HUVECs could be relieved by knockdown of FAIM2. To our knowledge, the current study first revealed that inhibition of *miR-193a-3p* could protect HUVECs against IH-induced injury by targeting FIAM2.

OSA, characterized by intermittent hypoxia, is considered as an independent risk factor for a variety of cardiovascular diseases, including myocardial ischemia, hypertension, atherosclerosis and heart failure [8, 22]. Multiple contributing factors supporting the potential association between OSA and cardiovascular diseases have been suggested, including intermittent hypoxia, oxidative stress, increased sympathetic activity, and systemic inflammation, all of which may be linked to endothelial dysfunction [8, 23, 24]. Endothelial dysfunction is an important onset in the pathogenesis of atherosclerosis and other cardiovascular disease [25, 26]. Studies have confirmed the association between OSA or IH and endothelial dysfunction [27–29]. IH during OSA leads to several pathological responses including oxidative stress and inflammation, which is suggested to account for endothelial dysfunction [30, 31]. In a word, consistent evidence shows that OSA may cause endothelial dysfunction. As yet, little is known about the processes leading from endothelial dysfunction to pathological changes of cardiovascular consequences in OSA. In our study, IH stimulation significantly reduced cell viability and promoted cell apoptosis in endothelial cells. Therefore, how to relieve IH-related endothelial injury arouses more and more concern.

In the past decade, a number of microRNAs have already been demonstrated to play crucial roles in the biological functions of endothelial cells (ECs), such as cell proliferation, migration, apoptosis, and differentiation [30–34]. Liu et al. disclosed that *miR-495* regulated the proliferation and apoptosis of HUVECs by directly targeting CCL2 [35]. Similarly, *miR-497* was identified to play an important role in the development of atherosclerosis by inducing apoptosis and suppressing the proliferation of HUVECs [36]. These all highlighted the critical role miRNAs involved in the apoptosis of ECs. Furthermore, miRNAs are also involved in the regulation of initiation and development of cardiovascular disease [37, 38]. For example, *miR-208* and *miR-1* are identified as novel biomarkers for cardiovascular diseases, including coronary artery disease, essential hypertension and heart failure [39]. Our results showed that *miR-193a-3p* was markedly upregulated after IH stimulation, implying the crucial function of *miR-193a-3p* in the progression of IH-induced endothelial injury. Therefore, we chose *miR-193a-3p* to explore the relationship between miRNAs and endothelial injury induced by IH in the present study.

To the best of our knowledge, our study is the first report about the function of *miR-193a-3p* in HUVECs under IH condition. It has now been revealed that *miR-193a-3p* plays a vital role in multiple diseases, such as acute myeloid leukemia [40], osteosarcoma cells [41] and colorectal cancer [42]. Recent studies have declared that *miR-193a-3p* participated in various biological processes, such as proliferation, migration, and apoptosis [43]. For example, *miR-193a-3p* overexpression can promote apoptosis and inhibit proliferation in H295R cells by targeting CYP11B2 [44]. Our study showed that *miR-193a-3p* inhibitor could reverse IH-induced apoptosis in HUVECs. Taken together, we identified that *miR-193a-3p* could mediate IH-induced endothelial injury in HUVECs.

To further clarify the mechanism of *miR-193a-3p* in HUVECs proliferation and apoptosis, we performed bioinformatic analysis and dual-luciferase reporter assay to find its target gene. By using miRbase, starBase, and TargetScan software, we found that 3′-UTR of FAIM2 contained the putative binding sites for *miR-193a-3p*. FAIM2, also called Lifeguard (LFG) or neural membrane protein 35 (NMP35), is an anti-apoptotic protein known as a distinct gene of the LFG family [45]. FAIM2 also takes part in other apoptotic-independent processes, such as axonal growth, neuronal differentiation, and neuroplasticity [46, 47]. Next, we demonstrated that overexpression of *miR-193a-3p* resulted in suppression of luciferase activity. In addition, we observed that *miR193a-3p* downregulation significantly increased mRNA and protein expression of FAIM2 in HUVECs under IH condition. Based on the above data, we indicated that FAIM2 is an important direct target of *miR-193a-3p* in HUVECs during IH. Finally, FAIM2 suppression could abolish the inhibitory effect of *miR-193a-3p* inhibitor on HUVECs proliferation and apoptosis under IH. In brief, our study first demonstrated that downregulation of *miR-193a-3p* attenuated IH-induced HUVECs injury by targeting FAIM2.

The goal of our study was only to assess the effect and potential mechanism of *miR-193a-3p* inhibition in vitro experiments just as a preliminary exploration. However, we must acknowledge that our study presents some limitations. Firstly, different stimulation times of IH is likely to show different effects on HUVECs, which requires to be further verified. Secondly, we did not investigate the morphological change of apoptosis. Thirdly, this study was conducted in vitro, therefore, more in vivo experiments are still needed to confirm the present observations in the future. Fourthly, we did not perform these experiments using a second human endothelial cell line or primary cells, which had to be considered as a disadvantage. Finally, other miRNAs and genes are likely to play critical roles in IH-induced endothelial injury. A single miRNA could regulate various target genes, and vice versa. Therefore, we will focus our attention on roles of other miRNAs and target genes on IH-induced endothelial injury in future studies.

Taken together, we confirmed that *miR-193a-3p* was increased in HUVECs under IH condition and *miR-193a-3p* inhibition could protect HUVECs from IH-induced injury. In addition, we first identified that *miR-193a-3p* down-regulation mediated IH-induced endothelial injury by regulating FAIM2 expression. Our findings will provide a novel understanding of the mechanism of IH-induced endothelial injury and thus serve as a potential therapeutic target for treating OSA-associated cardiac diseases.

## MATERIALS AND METHODS

### Cell culture

Human umbilical vein endothelial cells (HUVECs) was purchased from the Cell Bank of the Chinese Academy of Sciences(Shanghai, China). Cells were cultured in Dulbecco’s modified Eagle’s medium (HyClone) containing 10% fetal bovine serum (Gibco) and 1% penicillin/streptomycin, in a cell incubator with 5% CO2 at 37°C (Thermo, Waltham, MA, USA). HUVECs were found to be negative for mycoplasma by PCR to exclude the possibility of cryptic contamination.

### Establishment of IH model

When HUVECs were propagated to 70-80% confluence, the method of IH stimulation was carried out as previously described [48], with slight modifications. In brief, cells were maintained under hypoxia condition induced by flushing a mixed air of 1% O_2_ and 5% CO_2_ balanced with N_2_ for 35 min, and then normoxia condition (21% O_2_ with 5% CO_2_ balanced with N_2_ for 25 min). Repeated IH exposure was performed for 6 times.

### MiRNA target prediction

To predict the potential target genes of *miR-193a-3p*, three different miRNA target prediction algorithms: TargetScan7.2 (http://www.targetscan.org/), starBase (http://starbase.sysu.edu.cn/) and miRbase (http://www.mirbase.org/) were employed. Considering the high false positive rates of prediction, the three prediction tools were combined used to improve the quality of miRNA target prediction.

### Real-time quantitative PCR (RT-qPCR)

After intervention, mRNA of HUVECs was isolated using Trizol reagent (Takara) according to manufacturer’s protocol. To analyze the expression of *miR-193a-3p*, the RevertAidTM First Strand cDNA Synthesis Kit (#K1622; Thermo) with a special stem-loop primer and SYBR Green PCR Master Mix (#K0223; Thermo) were used to reverse transcription and quantitative PCR. To detect the expression level of FAIM2, the One Step SYBR^®^ PrimeScript^®^ PLUS RT-RNA PCR Kit (Takara) was applied. U6 and Actin were used as an internal control. The RT-qPCR was performed on ABI 7500 thermocycler (Applied Biosystems, Foster City, CA, USA). Each sample was measured in triplicate. Relevant primers were listed in the Table 1. The relative expression of qPCR results was calculated by the 2^−ΔΔCT^ method.

**Table 1:** Primers used for RT-qPCR.

**ID**	**Sequence (5′-3′)**
*miR-193a-3p*	Sense: ACACTCCAGCTGGGTGGGTCTTTGCGGGCG
	Antisense: TGGTGTCGTGGAGTCG
*miR-193a-3p* inhibitor	ACUGGGACUUUGUAGGCCAGUU
Inhibitor control	CAGUACUUUUGUGUAGUACAA
FAIM2	Sense: AGTTCGTCGAGTCTTTGTCAGA
	Antisense: GGGTCCAGAACAGCAAGC
si-FAIM2	Sense: GCGGGUGUAUUUACAUUGUTT
	Antisense: ACAAUGUAAAUACACCCGCTT
U6	Sense: CTCGCTTCGGCAGCACA
	Antisense: AACGCTTCACGAATTTGCGT
β-Actin	Sense: TGGACTTCGAGCAAGAGATG
	Antisense: TGTTGGCGTACAGGTCTTTG

### Cell transfection

*miR-193a-3p* inhibitor, small interfering RNA targeting FAIM2 (si-FAIM2), and corresponding scrambled control were chemically synthesized by Sangon Biotech Co.(Shanghai, China). When HUVECs in 6-well plates grew to about 80% confluence, we replaced the medium with serum-free medium. The cells were then transfected with *miR-193a-3p* inhibitor, si-FAIM2 and corresponding scrambled control using Lipofectamine 3000 (Invitrogen, USA) following manufacturer’s instructions. Cells were then exposed to IH.

### CCK-8 assay

The cell viability was detected by CCK-8 assay (TransGen Biotech, Beijing, China) following the manufacturer’s instructions. HUVECs were plated in 96-well (5 × 10^3^ cells/well). After IH stimulation, 10ul/well of CCK-8 was added into each well. Next, the mixture of 96-well plates was maintained at cell incubator for additional 2h. Finally, the absorbance was measured at 450nm with the use of a Multiskan GO Spectrophotometer (Thermo Fisher Scientific, USA).

### Western blot analysis

Proteins were extracted by using Mammalian Protein Extract on Reagent (CWBIO, Beijing, China) supplemented with protease inhibitors. Subsequently, BCA Protein Assay Kit (CWBIO, Beijing, China) was performed to determine protein concentrations. Equal amounts of protein were then separated by sodium dodecyl sulfate polyacrylamide gel electrophoresis (SDS-PAGE) and transferred to polyvinylidene fluoride (PVDF) membranes. Next, the membranes were blocked in 5% non-fat dry milk for 1 h, and then followed by incubation with primary antibodies at 4°C overnight. After washes, relevant secondary antibodies were applied at room temperature for 1 h. Finally, the membranes were washed and developed using standard chemiluminescence and the Bio-Rad ChemiDoc™ XRS+System. The intensity of bands was analyzed with Image-Pro Plus 6.0 software (Media Cybernetics, Rockville, MD, USA) and normalized to β-Actin.

### Dual-luciferase reporter assay

The fragment from FAIM2 3′-untranslated region (3′UTR), containing the predicted *miR-193a-3p* binding sequence, was amplified by PCR. To amplify the sequence for the mutation within the *miR-193a-3p* binding sites, we applied the point mutation method by using the KOD-Plus mutagenesis kit (Toyobo, Osaka, Japan). For dual-luciferase reporter experiments, the pSI-Check2 luciferase reporter vector containing the binding sites of 3′-UTR of FAIM2 mRNA or mutant 3′-UTR of FAIM2 was cotransfected with *miR-193a-3p* mimics or negative controls into HUVECs using Lipofectamine^TM^ 3000. After 48 h, we measured the firefly luciferase and renilla luciferase activity by a fluorescence detector (Promega). Renilla luciferase activities were normalized as control for each transfected well. Each experiment was replicated in triplicate.

### Statistics and data analysis

All statistical analyses were performed with SPSS 22.0 software. All data are presented as mean ± SD. Differences were compared by one-way analysis of variance, followed by a modified Student’s t test. Differences were considered statistically significant if p < 0.05. All experiments were repeated at least three times.
